# VFMAP predicted hepatocellular carcinoma development in patients with chronic hepatitis C who were treated with direct-acting antiviral and achieved sustained virologic response

**DOI:** 10.1007/s10396-023-01398-5

**Published:** 2023-12-26

**Authors:** Tomomitsu Matono, Toshifumi Tada, Takashi Nishimura, Tomoyuki Takashima, Nobuhiro Aizawa, Naoto Ikeda, Hideyuki Shiomi, Hirayuki Enomoto, Hiroko Iijima

**Affiliations:** 1 Department of Gastroenterology, Division of Hepatobiliary and Pancreatic Disease, Hyogo Medical University, 1-1 Mukogawacho, Nishinomiyashi, Hyogo 663-8501 Japan; 2 Department of Internal Medicine, Himeji St. Mary’s Hospital, Himeji, Hyogo Japan; 3 Department of Gastroenterology, Hyogo Prefectural Harima-Himeji General Medical Center, Himeji, Hyogo Japan; 4 Department of Internal Medicine, Japanese Red Cross Himeji Hospital, Hyogo, Japan; 5 Ultrasound Imaging Center, Hyogo Medical University, Hyogo, Japan

**Keywords:** Direct-acting antiviral, Sustained virological response, Hepatocellular carcinoma, VFMAP scoring system

## Abstract

**Purpose:**

Risk factors for the development of hepatocellular carcinoma (HCC) remain unclear in patients with hepatitis C virus (HCV) who achieve sustained virological response (SVR) after direct-acting antiviral (DAA) therapy. This study investigated the usefulness of the VFMAP scoring system for predicting the development of HCC in these patients.

**Methods:**

This study included 358 patients with HCV who achieved SVR after DAA treatment. The VFMAP system defines and scores cutoff values for virtual touch quantification (VTQ), fasting plasma glucose, sex, age, and alpha-fetoprotein values. All patients were grouped according to their VFMAP scores as follows: 0 or 1 point, low-score group; 2 or 3 points, intermediate-score group; and 4 or 5 points, high-score group.

**Results:**

Nineteen patients developed HCC. The median follow-up duration was 3.2 (1.5–4.0) years. The respective cumulative incidence rates of HCC at 12, 24, and 36 months were as follows in different subgroups: all study patients, 3.0%, 4.8%, and 6.6%; low-score group, 0.96%, 0.96%, and 0.96%; intermediate-score group, 2.6%, 4.5%, and 6.8%; and high-score group, 10.0%, 15.3%, and 18.5%. The cumulative incidence rates of HCC in the high-score group were significantly higher than those in the low- and intermediate-score groups (p < 0.001 and < 0.05, respectively).

**Conclusion:**

VFMAP accurately predicted the development of HCC in HCV patients who achieved SVR following treatment with DAAs.

## Introduction

Hepatitis C virus (HCV) infection affected 71 million people worldwide in 2015. Approximately 10–20% of patients with chronic HCV infection develop cirrhosis or hepatocellular carcinoma (HCC) [1]. In Japan, 1–1.5 million people are chronically infected with HCV, and approximately 55% of those with HCC have HCV infection [2].

Direct-acting antivirals (DAAs) have recently been developed for the treatment of chronic HCV infection. These drugs are associated with higher sustained viral response (SVR) rates, shorter and simpler treatment regimens, and fewer treatment-related side effects than conventional agents [3]. Several studies have shown that patients who achieve SVR with DAA therapy also have a lower incidence of HCC [4–6]. However, there is insufficient research on risk factors for the development of HCC in this population.

Advanced liver fibrosis is also a risk factor for the development of HCC [7]. Liver biopsy is still considered the gold standard for evaluating liver fibrosis even though it is painful, costly, and associated with limitations in diagnostic utility and accuracy. Moreover, since its invasiveness precludes repeated examinations [8], longitudinal evaluation of liver fibrosis is difficult. Transient elastography (TE; FibroScan, Echosens) is a noninvasive liver fibrosis test that is quick and easy to perform and is well accepted by patients. It has high accuracy and reproducibility when used to detect advanced fibrosis and cirrhosis. This noninvasive technique has made it possible to measure liver fibrosis in many patients. Acoustic radiation force impulse (ARFI) elastography [9–11] is a new ultrasonography (US)-based technique for noninvasively evaluating liver stiffness. This method can easily and accurately assess the degree of liver fibrosis in clinical practice [12, 13]. ARFI elastography has two modes: a qualitative response for virtual tissue imaging based on the area ratio and tissue displacement in the longitudinal direction, and a quantitative response for virtual touch quantification (VTQ) that measures transverse shear-wave velocity values in meters/second (m/s). Previously, we reported the usefulness of a US elastography–based scoring system combining VTQ, age, gender, fasting blood glucose, and alpha-fetoprotein (AFP) (VFMAP) for predicting liver carcinogenesis in patients with chronic liver disease [14].

In the present study, we investigated the usefulness of the VFMAP scoring system for predicting the development of HCC in patients who achieved SVR after DAA therapy.

## Materials and methods

### Patients

A total of 576 patients with HCV received DAA therapy at Hyogo College of Medicine between January 2013 and August 2020. Of these, 358 met the following eligibility criteria and were enrolled in this study: (1) completed an 8- or 12-week course of DAA therapy; (2) achieved SVR; (3) underwent VTQ assessment within 6 months after SVR; (4) underwent surveillance for HCC by blood tests (including tumor markers) and imaging with US, computed tomography (CT), and/or magnetic resonance imaging (MRI) at least every 3–6 months during the follow‐up period; (5) had no history of HCC recurrence within 1 year before the start of DAA therapy if there was a history of HCC; (6) had no evidence of HCC development or recurrence for at least 6 months after SVR; (7) had no co‐infection with human immunodeficiency virus or hepatitis B virus; (8) had no other causes of chronic liver disease (alcohol consumption more than 80 g/day, hepatotoxic drugs, autoimmune hepatitis, primary biliary cholangitis, hemochromatosis, and Wilson’s disease); and (9) had no missing clinical data.

In this study, patients with a history of HCC who experienced recurrence after SVR were required to have no evidence of HCC from 1 year before the start of DAA therapy to 24 weeks afterward (i.e., a total of over 1.5 years). Therefore, patients who had a history of HCC and who experienced recurrence after SVR were defined as equivalent to those who developed HCC for the first time and achieved SVR 24 weeks after DAA therapy.

The study protocol complied with the Helsinki Declaration and was approved by the institutional review board (#3825) of Hyogo College of Medicine. Before the start of the study, written informed consent was obtained from all patients for use of their laboratory data.

### Clinical and laboratory data

Patient age and sex were recorded 24 weeks after SVR on the same day as VTQ examinations. Serum samples were collected in the fasting state, and biochemistry tests were conducted using standard methods.

### VTQ

VTQ measurement by ARFI was performed with a Siemens ACUSON S2000 (Mochida Siemens Medical Systems, Tokyo, Japan). In this procedure, patients lie in the supine position with the right upper extremity lifted. The area of the liver to be examined for elastic properties is targeted with a region-of-interest (ROI) cursor while B-mode imaging is performed. Tissue in the ROI is mechanically excited using acoustic push pulses to generate localized tissue displacements that cause propagation of shear waves away from the region of excitation. These shear waves are tracked using ultrasonic correlation–based methods. The maximal displacement is estimated for many US tracking beams laterally adjacent to the single push beam. By measuring the time to peak displacement at each lateral location, the shear-wave propagation velocity can be reconstructed. In this study the examination was performed on the right lobe of the liver with a measurement depth of 2–3 cm below the liver capsule. Six successful acquisitions at different locations were performed on each patient; the median value was calculated, and the results are expressed in m/s. The shear-wave propagation velocity is considered to be proportional to the square root of tissue elasticity.

### Treatment

At baseline, HCV infection was confirmed by both positive serum HCV antibody titers (ARCHITECT Anti‐HCV; Abbott Laboratories) and serum HCV RNA using a real‐time PCR–based method (COBAS AmpliPrep/COBAS TaqMan HCV Test; Roche Molecular Systems; lower limit of detection, 1.2 log10IU/ml). At our hospital (Hyogo College of Medicine College Hospital), DAA therapy indications and regimens for each patient are determined according to the guidelines of the Japan Society of Hepatology.

SVR was confirmed by the absence of serum HCV RNA 24 weeks after the end of treatment (SVR24).

### HCC surveillance and diagnosis

In accordance with the Clinical Practice Guidelines for HCC in Japan [15], cirrhotic patients under surveillance underwent US (including VTQ examination) and monitoring of tumor markers every 3–4 months, and dynamic CT or MRI every 12 months. For patients with chronic hepatitis, we performed US (including VTQ examination) and monitoring of tumor markers every 6 months. As recommended by the diagnostic algorithm of the Japan Society of Hepatology, HCC was diagnosed principally based on the results of US and dynamic CT (hyperattenuation during the arterial phase in all or part of the tumor, and hypoattenuation in the portal venous phase) and/or MRI [15].

There were insufficient liver biopsy data to analyze liver fibrosis in this study; thus, it was assessed using the aspartate aminotransferase (AST)/platelet ratio index (APRI). This index was calculated as (AST [IU/l]/upper limit of normal AST [IU/l]) × 100/platelet count [10^9^/l], and its clinical utility in diagnosing liver fibrosis has been previously reported [16].

### Definitions of parameter cutoff values and VFMAP groups

We defined cutoff values for each parameter according to previously published reports: high age, ≥ 55 years; low albumin level, ≤ 4.2 g/dl; high AFP level, ≥ 5 ng/ml; fasting hyperglycemia, fasting plasma glucose (FPG) level ≥ 110 mg/dl; high VTQ value, > 1.33 m/s.

The VFMAP scoring system combines the VTQ value, FPG, sex, age, and AFP level. VTQ values of ≤ 1.33 and > 1.33 m/s were scored as 0 and 1, respectively. FPG levels < 110 and ≥ 110 mg/dl were scored as 0 and 1, respectively. Female and male sex were scored as 0 and 1, respectively. Age < 55 and ≥ 55 years were scored as 0 and 1, respectively. AFP levels < 5 and ≥ 5 ng/ml were scored as 0 and 1, respectively. The total score was defined as the sum of the VTQ, FPG, sex, age, and AFP scores. All patients were grouped based on their VFMAP scores as follows: 0 or 1 point, low-score group; 2 or 3 points, intermediate-score group; and 4 or 5 points, high-score group.

### Statistical analysis

Continuous variables are expressed as medians (first–third quartiles). The Mann–Whitney *U* test was used to compare continuous variables, and the χ^2^‐test or Fisher’s exact test was used for categorical variables.

Actuarial analysis of cumulative overall survival was performed using the Kaplan–Meier method, and differences were assessed with the log‐rank test. A univariate Cox proportional hazards model was used to analyze the incidence of HCC.

The Kaplan–Meier method and a univariate Cox proportional hazards model were first used to evaluate the cumulative incidence of the HCC rate, both in the overall study population and in the populations stratified by low, intermediate, and high VFMAP scores.

Statistical significance was defined as p < 0.05. Statistical analyses were carried out with Easy-R (EZR) version 1.55 (Saitama Medical Center, Jichi Medical University, Saitama, Japan), which is a graphical user interface for R (The R Foundation for Statistical Computing, Vienna, Austria). More precisely, it is a modified version of the R commander designed to add statistical functions frequently used in biostatistics.

## Results

### Patient characteristics and survival

The characteristics of the 358 patients are shown in Table 1. The patients included 205 (57.3%) women and 153 (42.7%) men, with a median age of 67.0 years (57.0–74.0 years). Fifty-four (15.1%) patients had a history of HCC before the start of DAA therapy. The median VTQ was 1.23 m/s (1.08–1.58 m/s), the median fasting plasma glucose was 102 mg/dl (95–113 mg/dl), and the median AFP level was 3.4 ng/ml (2.2–5.0 ng/ml). Nineteen patients developed HCC. The median follow-up period was 3.15 years (1.51–3.97 years).

**Table 1:** Patient characteristics (n = 358)

Age (years)	67.0 (57.0–74.0)
Sex (female/male)	205/153
History of HCC (yes/no)	54/304
Platelet count (× 10^4^/mm^3^)	16.6 (12.6–21.2)
Prothrombin time (%)	91.0 (81.8–100.2)
Total bilirubin (mg/dl)	0.7 (0.6–1.0)
Albumin (g/dl)	4.2 (4.0–4.5)
Aspartate aminotransferase (IU/L)	22 (18–27)
Alanine aminotransferase (IU/L)	15 (11–20)
Fasting plasma glucose (mg/dl)	102 (95–113)
Alpha-fetoprotein (ng/ml)	3.4 (2.2–5.0)
Virtual touch quantification (m/s)	1.23 (1.08–1.58)
DAA (daclatasvir + asunaprevir/ledipasvir‐sofosbuvir/ombitasvir‐paritaprevir‐ritonavir/sofosbuvir + ribavirin/elbasvir‐grazoprevir/glecaprevir‐pibrentasvir/clinical trial)	81/137/17/57/14/42/10
Developed HCC	19
Recurrence history of HCC (yes/no)	11/8
Follow-up period (years)	3.15 (1.51–3.97)

### Cumulative incidence of HCC

**Fig. 1:**
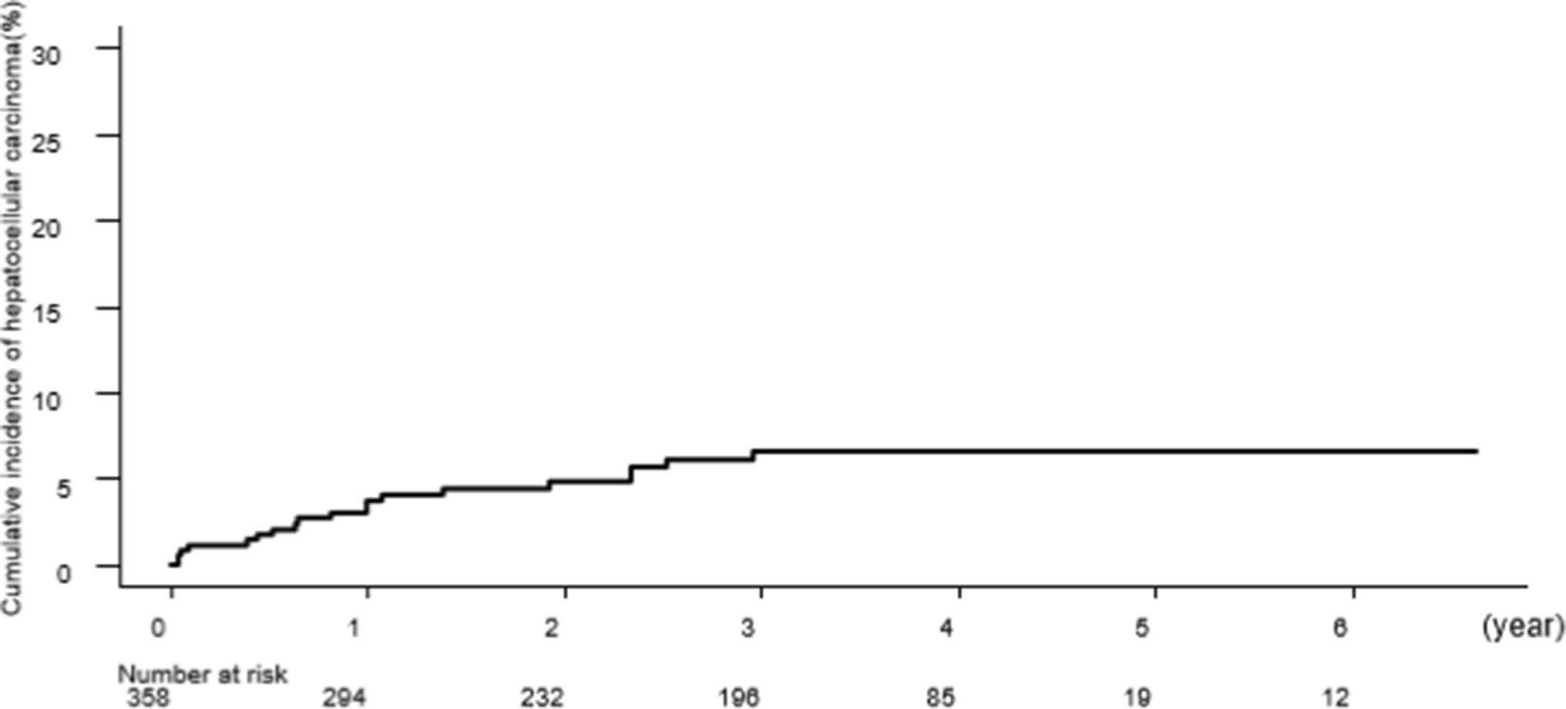
Cumulative incidence rates of hepatocellular carcinoma. The 12-, 24-, and 36-month cumulative incidence rates of HCC were 3.0%, 4.8%, and 6.6%, respectively

Figure 1 shows the Kaplan–Meier curve for the cumulative incidence of HCC in all study patients. The 12-, 24-, and 36-month cumulative incidence rates of HCC were 3.0%, 4.8%, and 6.6%, respectively.

### Characteristics and cumulative incidences of HCC according to VFMAP scores

**Fig. 2:**
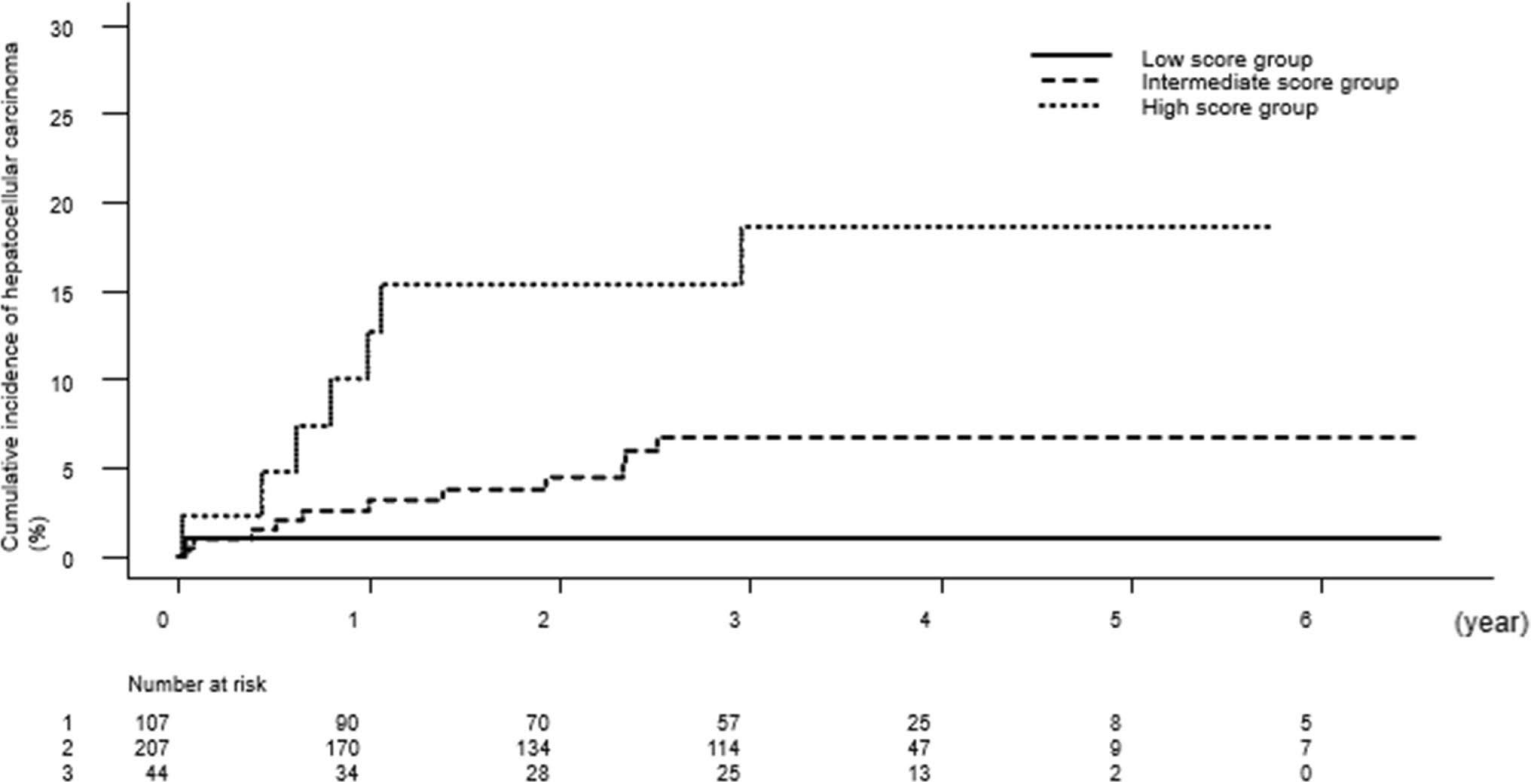
The respective Kaplan–Meier curves for the 12-, 24-, and 36-month cumulative incidence rates of hepatocellular carcinoma according to subgroup: in the low-score group, 0.96%, 0.96%, and 0.96%; in the intermediate-score group, 2.6%, 4.5%, and 6.8%; and in the high-score group, 10.0%, 15.3%, and 18.5%

Table 2 shows the characteristics of patients according to VFMAP scores. There were significant differences between the low-, intermediate- and high-score groups for all characteristics. Figure 2 shows the respective Kaplan–Meier curves for the 12-, 24-, and 36-month cumulative incidence rates of HCC according to subgroup: in the low-score group, 0.96%, 0.96%, and 0.96%; in the intermediate-score group, 2.6%, 4.5%, and 6.8%; and in the high-score group, 10.0%, 15.3%, and 18.5%. The cumulative incidence rates of HCC in the high-score group were significantly higher than those in the low- and intermediate-score groups (p < 0.001 and < 0.05, respectively).

**Table 2:** Patient characteristics between VFMAP groups

	Low-score group n = 107	Intermediate-score group n = 207	High-score group n = 44	
Age (years)	63.0 (52.0–71.5)	69.0 (60.0–75.0)	67.0 (61.0–71.3)	< 0.001
Sex (female/male)	88/19	109/98	8/36	< 0.001
History of HCC (yes/no)	4/103	37/170	13/31	0.001
Platelet count (× 10^4^/mm^3^)	19.4 (16.1–23.8)	15.9 (12.4–20.5)	11.3 (8.7–16.6)	< 0.001
Prothrombin time (%)	93.6 (84.8–100.9)	90.0 (81.4–100.2)	89.4 (77.5–96.3)	n.s
Total bilirubin (mg/dl)	0.7 (0.5–0.9)	0.8 (0.6–1.0)	0.9 (0.5–1.2)	< 0.05
Albumin (g/dl)	4.3 (4.1–4.5)	4.2 (4.0–4.5)	4.1 (3.8–4.3)	< 0.05
Aspartate aminotransferase (IU/L)	20 (93–102)	23 (19–27)	23 (18–30)	< 0.05
Alanine aminotransferase (IU/L)	13 (11–17)	15 (11–20)	19 (15–24)	< 0.001
Fasting plasma glucose (mg/dl)	98 (93–102)	103 (95–114)	123 (112–134)	< 0.001
Alpha-fetoprotein (ng/ml)	2.5 (1.9–3.6)	3.7 (1.5–4.0)	5.6 (3.5–7.7)	< 0.001
virtual touch quantification (m/s)	1.09 (1.02–1.18)	1.33 (1.11–1.62)	1.85 (1.55–2.17)	< 0.001
DAA (daclatasvir + asunaprevir/ledipasvir‐sofosbuvir/ombitasvir‐paritaprevir‐ritonavir/sofosbuvir + ribavirin/elbasvir‐grazoprevir/glecaprevir‐pibrentasvir/clinical trial)	18/41/6/20/6/14/2	48/80/10/29/8/26/6	15/16/1/8/0/2/2	n.s
Developed HCC	1	11	7	0.001
Follow-up period (years)	3.15 (1.59–3.92)	3.15 (1.45–3.95)	3.19 (1.05–4.08)	n.s

n.s.: not significant

Multivariate analysis with Cox proportional hazards modeling showed that the VFMAP score (evaluated at 1-point intervals) and a history of HCC were significantly associated with HCC development (hazard ratio [HR] 1.84, 95% confidence interval [CI] 1.14–2.97, p = 0.012; and HR 4.54, 95% CI 1.70–12.08, p = 0.003, respectively).

## Discussion

DAAs have recently been introduced as simple and safe oral antiviral drugs for HCV infection, and have been reported to reduce the risk of HCC [17, 18]. Kurosaki et al. [19] reported a model to detect HCV patients at high risk of HCC, and found that the incidence of HCC was reduced more significantly in high- and intermediate-risk patients who achieved SVR with interferon (IFN)-based therapy than in non-SVR patients. Thus, establishing a scoring system based on common clinical data to predict HCC development in HCV patients who have achieved SVR with DAA therapy is an important issue in an era when SVR after DAA therapy is relatively common.

Previous reports have shown that the FIB-4 index is an indicator of advanced fibrosis [20, 21], and that an elevated FIB-4 index is a risk factor for HCC in patients who have achieved SVR with IFN-based therapy [22]. Also, in an analysis of the impact of liver fibrosis on the development of HCC using FibroScan, advanced fibrosis (i.e., higher TE values) was associated with a higher incidence of HCC in HCV patients who had achieved SVR with DAA therapy [23]. Thus, an index of liver fibrosis is considered to be an independent prognostic predictor in post-SVR HCV patients.

Freidrich-Rust et al. [12] conducted a meta-analysis of nine studies of patients with chronic liver disease to evaluate the diagnostic performance of ARFI in the staging of liver fibrosis, and reported that ARFI had good diagnostic accuracy for liver fibrosis and excellent accuracy for cirrhosis. Several recent reports have also shown that liver stiffness measurements using US elastography are useful for predicting the development of HCC [24–28].

On the other hand, elevated serum AFP levels have also been reported as a risk factor for HCC development in HCV-infected patients, with age, male sex, FIB-4 index at SVR, and AFP levels extracted as predictors of worse HCC prognosis [7, 29–31, 31–34]. Koh et al. [35] analyzed a large number of patients with long-term follow-up and found that a history of diabetes was also associated with the risk of developing HCC. Based on these reports, to predict the development of HCC in patients with chronic liver disease, we previously established a new scoring system, VFMAP, based on older age, male sex, liver fibrosis (VTQ), high AFP level, and fasting hyperglycemia. In an analysis of patients stratified by VFMAP score, the high-score group had a higher hazard ratio than the low-score group, indicating that VFMAP accurately predicts the development of HCC in patients with chronic liver disease [14]. The VFMAP system includes all parameters previously associated with the risk of developing HCC, and is more accurate and convenient for predicting HCC risk than prior methods.

In this study, we tested whether the VFMAP scoring system could predict the development of HCC in HCV patients who achieved SVR after DAA treatment. HCC was significantly more likely to develop in the high-score group (score 4 or 5) than in the medium-score group (2 or 3) and the low-score group (0 or 1), with the cumulative incidence rates of HCC in the high-score group being 10%, 15.3%, and 18.5% at 1, 2, and 3 years, respectively. These results indicate that the VFMAP scoring system accurately predicts the development of HCC in patients with HCV who were treated with DAAs and subsequently achieved SVR.

This study had several limitations. It used a retrospective, single-center design, and additional studies with larger numbers of patients and participating centers are needed. Eight cases were patients in whom HCC occurred for the first time and 11 cases were patients who experienced recurrence. Therefore, it was necessary to consider carcinogenesis due to first occurrence and recurrence separately in this study. However, due to the small number of patients who developed HCC (54 cases), it was difficult to analyze statistically. In addition, the median observation period was only 3.15 years. Therefore, a longer observation period is needed in the future.

## Conclusion

The VFMAP scoring system, based on VTQ, fasting hyperglycemia, sex, age, and AFP level, could accurately predict the development of HCC in HCV patients who achieved SVR after DAA therapy. It will be important to validate this scoring system in further studies with other populations.

## Data Availability

The datasets generated and/or analyzed during the current study are available from the corresponding author on reasonable request.

## Abbreviations

HCV: Hepatitis C virus
HCC: Hepatocellular carcinoma
DAA: Direct-acting antiviral
SVR: Sustained viral response
TE: Transient elastography
US: Ultrasonography
ARFI: Acoustic radiation force impulse
VTQ: Virtual touch quantification
CT: Computed tomography
MRI: Magnetic resonance imaging
AST: Aspartate aminotransferase
APRI: Aspartate aminotransferase/platelet ratio index
FPG: Fasting plasma glucose
AFP: Alpha-fetoprotein

## Declarations

### Registry and the registration No. of the study

The institutional review board (#3825) of Hyogo College of Medicine.
