# Correction: VFMAP predicted hepatocellular carcinoma development in patients with chronic hepatitis C who were treated with direct-acting antiviral and achieved sustained virologic response

**DOI:** 10.1007/s10396-025-01537-0

**Published:** 2025-04-06

**Authors:** Tomomitsu Matono, Toshifumi Tada, Takashi Nishimura, Tomoyuki Takashima, Nobuhiro Aizawa, Naoto Ikeda, Hideyuki Shiomi, Hirayuki Enomoto, Hiroko Iijima

**Affiliations:** 1 Department of Gastroenterology, Division of Hepatobiliary and Pancreatic Disease, Hyogo Medical University, 1-1 Mukogawacho, Nishinomiyashi, Hyogo 663-8501 Japan; 2 Department of Internal Medicine, Himeji St. Mary’s Hospital, Himeji, Hyogo Japan; 3 Department of Gastroenterology, Hyogo Prefectural Harima-Himeji General Medical Center, Himeji, Hyogo Japan; 4 Department of Internal Medicine, Japanese Red Cross Himeji Hospital, Hyogo, Japan; 5 Ultrasound Imaging Center, Hyogo Medical University, Hyogo, Japan

**Correction: Journal of Medical Ultrasonics (2023) 51:293–300** 10.1007/s10396-023-01398-5

The article “VFMAP predicted hepatocellular carcinoma development in patients with chronic hepatitis C who were treated with direct-acting antiviral and achieved sustained virologic response”, written by Tomomitsu Matono, Toshifumi Tada, Takashi Nishimura, Tomoyuki Takashima, Nobuhiro Aizawa, Naoto Ikeda, Hideyuki Shiomi, Hirayuki Enomoto and Hiroko Iijima, was originally published under exclusive license to The Author(s), under exclusive licence to The Japan Society of Ultrasonics in Medicine. As a result of the subsequent decision to publish the article under the open access model, the article’s copyright notice was changed on 18 March 2025 to © The Author(s) 2023 and the article is now distributed under a Creative Commons Attribution 4.0 International License, which permits use, sharing, adaptation, distribution and reproduction in any medium or format, as long as you give appropriate credit to the original author(s) and the source, provide a link to the Creative Commons licence, and indicate if changes were made. The images or other third party material in this article are included in the article’s Creative Commons licence, unless indicated otherwise in a credit line to the material. If material is not included in the article’s Creative Commons licence and your intended use is not permitted by statutory regulation or exceeds the permitted use, you will need to obtain permission directly from the copyright holder. To view a copy of this licence, visit http://creativecommons.org/licenses/by/4.0

The original article has been corrected.

